# nlive: an R Package to facilitate the application of the sigmoidal and random changepoint mixed models

**DOI:** 10.21203/rs.3.rs-2235106/v1

**Published:** 2023-04-10

**Authors:** Ana W. Capuano, Maude Wagner

**Affiliations:** 1 RUSH Alzheimer’s Disease Center, Rush University Medical Center, 1750 Harrison Chicago, IL 60612, USA; 2 Bordeaux University, 146 rue Léo-Saignat, Bordeaux, FR

**Keywords:** longitudinal outcome, nlive, non-linear mixed model, random changepoint model, R package, saemix, sigmoidal mixed model, stochastic approximation expectation maximization

## Abstract

**Background:**

The use of mixed effect models with a specific functional form such as the Sigmoidal Mixed Model and the Piecewise Mixed Model (or Changepoint Mixed Model) with abrupt or smooth random change allow the interpretation of the defined parameters to understand longitudinal trajectories. Currently, there are no interface R packages that can easily fit the Sigmoidal Mixed Model allowing the inclusion of covariates or incorporate recent developments to fit the Piecewise Mixed Model with random change.

**Results:**

To facilitate the modeling of the Sigmoidal Mixed Model, and Piecewise Mixed Model with abrupt or smooth random change, we have created an R package called nlive. All needed pieces such as functions, covariance matrices, and initials generation were programmed. The package was implemented with recent developments such as the polynomial smooth transition of piecewise mixed model with improved properties over Bacon-Watts, and the stochastic approximation expectation-maximization (SAEM) for efficient estimation. It was designed to help interpretation of the output by providing features such as annotated output, warnings, and graphs. Functionality, including time and convergence, was tested using simulations. We provided a data example to illustrate the package use and output features and interpretation. The package implemented in the R software is available from the Comprehensive R Archive Network (CRAN) at https://CRAN.R-project.org/package=nlive.

**Conclusions:**

The nlive package for R fits the Sigmoidal Mixed Model and the Piecewise Mixed: abrupt and smooth. The nlive allows fitting these models with only five mandatory arguments that are intuitive enough to the less sophisticated users.

## Background

Continuous longitudinal data may have a trajectory that is not linear. This is the case in the study of cognitive aging, which presents a faster decline close to death, as well as the process in many other fields such as agriculture [1], pharmacology [2] and marketing [3]. Although some less parsimonious models have been proposed to model such longitudinal data, the use of models with a specific functional form such as the Sigmoidal Mixed Model (SMM) [4] and the Piecewise Mixed Model (PMM) [5] with abrupt or smooth change allow the interpretation of the defined parameters.

The SMM is currently implemented in SAS using PROC NLMIXED [4], which maximizes the marginal likelihood by using an adaptive Gaussian quadrature [6] or other approximation methods, such as the first-order method [7]. However, none of these packages can fit the SMM allowing the inclusion of covariates for all 4 parameters. The PMM is commonly fitted using Bayesian inference and implemented in OpenBugs or WinBUGS, but is also commonly fit in R using the lme4 [8], which maximizes the marginal likelihood by using a Laplace approximation. A recently developed Stochastic Approximation Expectation Maximization (SAEM) algorithm was shown to be more successful [9] and faster [10] to identify the maximum likelihood estimators of non-linear mixed models. This can be implemented directly using the package saemix [11] (version 3.0). The limitation is that both lme4 and saemix are very flexible packages that can fit a wide range of models and because of that they require more analytical skills as they are not a one-line of code. It is worth noting that there are some simple-to-use packages in R that can fit the abrupt PMM, including segmented [12] and rcpm [13]. However, these packages also do not use SAEM and with them, it is not possible to (i) include covariates for all 4 parameters, (ii) consider a smooth polynomial transition, and/or (iii) estimate directly the last level (e.g. level close to death).

In this work, we present the *nlive* package implemented within R software. The main objective of the package is to facilitate and broaden the application and interpretation of the SMM and PMM for longitudinal data. All needed elements to fit the models have been programmed, including the computation of the structural model and the automatic generation of initials for the main parameters. As such, less experienced R users only need to specify the model to fit via a single intuitive line of code, with only five mandatory arguments. The package was implemented with the most recent and efficient algorithms for non-linear models. Implementation was also performed with the most interpretable parameterization and was based on the most recent developments in each type of model. For example, for the smooth PMM, instead of using the Bacon–Watts [14] which can create an artificial increase in the trajectory right after the changepoint [15], we considered the most recently developed polynomial smooth transition [16]. In the following, we reintroduce these models, describe the implementation of the package, and provide a simulation study to demonstrate the performance of the package. We also demonstrate the use of the model and interpretation of the output using a made-up illsutrative sample dataset with trajectories similar to those observed in cohorts such as the Religious Order Study and the Rush Memory and Aging Project [17].

## Model specifications

As a prelude to the introduction and demonstration of the new *nlive* package, we first describe the general formulation of the nonlinear mixed models implemented in the package. The simplified general form of nonlinear mixed models can be written in terms of a known nonlinear function *f* given by:

(1) $$y_{ij}=f\left(t_{ij},\psi_{i}\right)+\epsilon_{ij}$$

where $y_{ij}$ denotes the longitudinal outcome value of subject *i* (i=1,…,N) collected at the observation time $t_{ij}$ $\left(j=1,\ldots ,n_{i}\right)$; $\psi_{i}$ is a vector of normally distributed person-specific parameters function of fixed effects and individual random effects; and $\epsilon_{ij}$ are random error, with $\epsilon_{ij}∼N\left(0,\sigma_{\epsilon }^{2}\right)$.

Motivated by the application on late-life cognitive decline, the *nlive* package implements two main classes of nonlinear mixed models: the Sigmoidal Mixed Model (SMM) [18, 4] with four parameters and the Piecewise Linear Mixed Model (PMM) [5] with two linear phases and a single changepoint. In the following sub-sections, we provide a brief introduction of these models. For simplicity, some annotations can be similar from one model to another, while the interpretation of the parameters remains specific to each of them.

### The Sigmoidal Mixed Model

The SMM introduced by Capuano and colleagues [4] is based on the four-parameter logistic that allows the inclusion of covariates related to four parametric quantities. The non-linear trajectory of the outcome *Y* can be formulated as follows:

(2) $$f\left(t_{ij},\psi_{i}\right)=\psi_{1i}+\frac{\psi_{2i}-\psi_{1i}}{1+{\left(t_{ij}/\psi_{3}\right)}^{\psi_{4}}}$$

where the first parameter, $\psi_{1i}$, represents the person-specific initial level of the outcome before the onset of decline. The second parameter, $\psi_{2i}$, represents the person-specific level of the outcome at time equal to zero (e.g., death), or the intercept. We will call it the last level although the meaning of time may differ depending on the application. $\psi_{3}$ represents the marginal time when half of the total decline occurred. We will call it the midpoint. $\psi_{4}$ represents the marginal Hill slope and will define the nonlinear pattern of the trajectory (e.g. determining the steepness, earlier versus later acceleration of change). These two latter parameters are kept as marginal for convergence purposes [4]. The four parameters are assumed to obey the following equations:

(3) $$\text{initial level}:\psi_{1i}=\alpha_{1}+\beta_{1}X_{1i}+\eta_{1i}$$

(4) $$\text{last level}\left(\text{intercept}\right):\psi_{2i}=\alpha_{2}+\beta_{2}X_{2i}+\eta_{2i}$$

(5) $$\text{midpoint or time of half decline}:\psi_{3}=\alpha_{3}+\beta_{3}X_{3i}$$

(6) $$\text{Hill slope}:\psi_{4}=\alpha_{4}+\beta_{4}X_{4i}$$

where $\alpha_{1}$, $\alpha_{2}$, $\alpha_{3}$, and $\alpha_{4}$ are the mean values for the last level, initial level, midpoint, and Hill slope, respectively; *X*_1*i*_, *X*_2*i*_, *X*_3*i*_, and *X*_4*i*_ are vectors of covariates associated with the vector of fixed effects $\beta_{1}$, $\beta_{2}$, $\beta_{3}$, and $\beta_{4}$, respectively; and $\eta_{1i}$ and $\eta_{2i}$ are random effects with ${\left(\eta_{1i},\eta_{2i}\right)}^{⊤}∼MVN\left(0,B\right)$ and *B* assuming correlations between $\eta_{1i}$ and $\eta_{2i}$.

### The Piecewise Linear Mixed Model with a Random Changepoint

The PMM model [5] assumes that the stochastic process of the longitudinal outcome is characterized by two or more different phases. Under this class of models, the *nlive* package implements two PMM models with an abrupt change (PMM-abrupt) [19] and a smooth polynomial transition (PMMs-mooth) [16] between the two linear phases. These models provide an appealing statistical approach to detect the time when the onset of accelerated decline occurs.

#### PMM with abrupt change

The PMM-abrupt model (also known as the linear-linear or the broken-stick mixed model), consists of an intercept at time zero, a slope close to the intercept, a change point at which the slope changes, and a slope after this change point. The non-linear trajectory of the outcome *Y* can be formulated as follows:

(7) $$f\left(t_{ij},\psi_{i}\right)=\{\begin{array}{ll} \psi_{1i}+\psi_{2i}\psi_{4i}+\psi_{3i}\left(t_{ij}-\psi_{4i}\right) & \text{if}\;t_{ij}<\psi_{4i}\\ \psi_{1i}+\psi_{2i}t_{ij} & \text{if}\;t_{ij}\geq \psi_{4i} \end{array}$$

where the first parameter, $\psi_{1i}$, represents the person-specific level of the outcome at time zero, or the intercept; $\psi_{2i}$ represents the person-specific slope before the changepoint; $\psi_{3i}$ represents the person-specific slope after the changepoint; and $\psi_{4i}$ represents the person-specific changepoint time parameter.

Assuming an alignment at death for example (for interpretation purposes), the parameters $\psi_{1i}$ to $\psi_{4i}$ are supposed to obey the following equations:

(8) $$\text{last level}\;\left(\text{intercept}\right):\psi_{1i}=\alpha_{1}+\beta_{1}X_{1i}+\eta_{1i},$$

(9) $$\text{slope before the changepoint}:\psi_{2i}=\alpha_{2}+\beta_{2}X_{2i}+\eta_{2i},$$

(10) $$\text{slope after the changepoint}:\psi_{3i}=\alpha_{3}+\beta_{3}X_{3i}+\eta_{3i},$$

(11) $$\text{changepoint time}:\psi_{4i}=\alpha_{4}+\beta_{4}X_{4i}+\eta_{4i}$$

where $\alpha_{1}$, $\alpha_{2}$, $\alpha_{3}$, and $\alpha_{4}$ are the mean values for the last level, the slope before the change point, the slope after the changepoint, and the changepoint time, respectively; *X*_1*i*_, *X*_2*i*_, *X*_3*i*_, and *X*_4*i*_ are vectors of covariates associated with the vector of fixed effects $\beta_{1}$, $\beta_{2}$, $\beta_{3}$, and $\beta_{4}$, respectively; and $\eta_{1i}$ to $\eta_{4i}$ are random effects with ${\left(\eta_{1i},\eta_{2i},\eta_{3i},\eta_{4i}\right)}^{⊤}∼MVN\left(0,B\right)$ and *B* assuming correlations only between $\eta_{2i}$ and $\eta_{3i}$.

### PMM with smooth polynomial transition

The PMM-smooth model is an extension of the abrupt PMM. The initial smooth PMM was proposed by Bacon and Watts [14] and included a hyperbolic tangent transition. In this work, however, we consider a more recent development that considers a smooth polynomial transition introduced in Van den Hout, Muniz-Terrera, and Matthews [16]. Unlike the Bacon-Watts model, the PMM-smooth enables a direct interpretation of the parameters associated with the two linear phases and allows to model the beginning of the smooth transition – that is the onset of the changepoint – ather than the middle of the smooth transition in the Bacon-Watts model.

In PMM-smooth, the transition is modeled using a third-degree polynomial function fitted between the two straight lines. In the original work [16, 20], the intercept parameter cannot be interpreted directly as it reflects the level parameter projection using the early slope at time zero. To allow direct interpretation of the intercept, we re-formulated the PMM-smooth model as:

(12) $$f\left(t_{ij},\psi_{i}\right)=\{\begin{array}{ll} \psi_{1i}+\psi_{2i}t_{ij}+\left(\psi_{3i}-\psi_{2i}\right)\left(t_{ij}-\psi_{4i}+\frac{v}{2}\right) & \text{if}\;t_{ij}<\psi_{4i}\\ g_{transition}\left(t_{ij}|\psi_{1i},\psi_{2i},\psi_{3i},v\right) & \text{if}\;\psi_{4i}\leq t_{ij}\leq \psi_{4i}+v\\ \lambda +\psi_{2i}t_{ij} & \text{if}\;t_{ij}<\psi_{4i}+v \end{array}$$

where $\psi_{1i}$, $\psi_{2i}$, and $\psi_{3i}$ have been previously defined for Equation (7). $\psi_{4i}$ is the person-specific time when the smooth transition phase of length *v* begins. *v* is a value representing the time interval where the polynomial curve occurs between $t_{ij}=\psi_{4i}$ and $t_{ij}=\psi_{4i}+v$. In order to be closer to the PMM-abrupt, the two linear parts should intersect at the middle of the transition phase and the constraint $\lambda_{i}=\psi_{1i}+\psi_{2i}\left(\psi_{4i}+\frac{v}{2}\right)-\psi_{3i}\left(\psi_{4i}+\frac{v}{2}\right)$ is imposed. Note that *v* set to 0 reduces to a PMM-abrupt model.

The smoothness of the transition function involves four linear equations with four parameters:

(13) $$g_{transition}\left(\psi_{4i}\right)=\lambda_{i}+\psi_{3i}\psi_{4i}$$

(14) $$g_{transition}\left(\psi_{4i}+v\right)=\psi_{1i}+\psi_{2i}\left(\psi_{4i}+v\right)$$

(15) $$\left(\frac{\partial }{\partial t_{ij}}g_{transition}\right)\left(\psi_{4i}\right)=\psi_{3i}$$

(16) $$\left(\frac{\partial }{\partial t_{ij}}g_{transition}\right)\left(\psi_{4i}+v\right)=\psi_{2i}$$

where *g_transition_* is obtained by solving the system of four linear equations with four unknown parameters. The derivatives of *g*_*transition*_ at the times $t_{ij}=\psi_{4i}$ and $t_{ij}=\psi_{4i}+v$ are respectively $\psi_{3i}$ and $\psi_{2i}$.

## Implementation

### Software

To facilitate the application and interpretation of the SMM, PMM-abrupt, and PMM-smooth models for a broader audience, who is not necessarily familiar with statistical programming, we developed a user-friendly R package called “nlive” (**n**on-**l**inear mixed models with **i**nitial **v**alues **e**stimated) with R version 4.0.3. All needed elements to fit the models, including the definition of the structural model and the generation of initials for the four main parameters, have been programmed so that the user only need to specify a single intuitive line of code to obtain estimations in both tabular and graphical formats. A variety of options can also be specified.

The *nlive* package was built on top of several existing R packages and is freely available via the Comprehensive R Archive Network (CRAN) at https://CRAN.R-project.org/package=nlive. A made-up dataframe, under the name “dataset”, is provided with the package and was generated to mimic cognitive decline patterns observed before death in the Religious Order Study and the Memory and Aging Project (ROSMAP) from the Rush Alzheimer’s Disease Center [17]; this dataset is used in the Example Section.

### Estimation

The SMM, PMM-abrupt, and PMM-smooth previously described were all estimated using the *saemix* package (version 3.0) developed by Comets and colleagues [11]. The *saemix* package, among other things, implies the definition of the structural model; thus, first, we programmed the structures of the models. For SMM, we relied on the *SSlogis*5() function of the *nlraa* package [21] (version 1.2), which initially defines a 5-parameter logistic curve but can be reduced to a 4-parameter logistic when the 5^*th*^ parameter is fixed to 1. For PMM-abrupt and PMM-smooth, since the specification of these models was not available in any existing package, we coded it explicitly. Note that, as *saemix* only provides p-values for the effects of the covariates, and not for the main parameters, the calculation of these p-values has also been programmed.

### The SAEM algorithm

The computational technique for maximum likelihood estimation implemented in *saemix* is the Stochastic Approximation Expectation Maximization (SAEM) algorithm, which is a stochastic approximation version of the standard EM algorithm proposed by Khuhn and Lavielle [22]. The SAEM algorithm showed to be efficient in the context of non-linear mixed models, converging quickly to the maximum likelihood estimators [10] and achieving better performance than linearization-based algorithms [9]. In preliminary testing during the algorithm coding process, in line with the literature, *saemix* showed convergence to the adequate solution more often than two main competing software package [11]: *nlme* [6] and lme4 [8].

### Initial values

The SAEM algorithm requires that the four main parameters of the models are specified, namely ${\hat{\alpha }}_{1}$, ${\hat{\alpha }}_{2}$, ${\hat{\alpha }}_{3}$, and ${\hat{\alpha }}_{4}$. By choosing values close to the maximizer, the number of iterations needed to reach convergence should be reduced. Thus, in *nlive*, for each model, we have designed an algorithm that generates informative initial values informed by the input dataset.

For SMM, the four main parameters to be specified reflect the last level, first level, midpoint, and Hill slope. These starting values were automatically computed building upon the algorithm developed in SAS by Capuano and colleagues [4] (algorithm hosted and accessible at github.com/AWCapuano/sigmoidal). This SAS algorithm was based on the original motivating case of longitudinal cognitive data and we generalized it here for other types of data (e.g., different time scales). Briefly, the initial and final levels of the outcome are informed by the average levels observed at the 5^*th*^ and 95^*th*^ percentiles of the time distribution. The time of half decline is set to 300 if the curve is nearly linear, and to 2 otherwise. Finally, the Hill slope is set to a high and low value based (0.5 and 1.05).

Similarly, for PMM-abrupt and PMM-smooth, estimation of the models requires the specification of four starting values related to the four main parameters: last level, slope before the changepoint, slope after the changepoint, and the changepoint time. The last level is informed by the mean level observed at the 95th percentile of the time distribution. For the other three parameters, we first estimate where the acceleration of decline (i.e., changepoint) occurred approximately over time by estimating five separate standard linear mixed models, each considering a subset of cognitive measures collected every 20 percentiles of the time distribution (i.e., [0, 20th percentile[, [20th percentile[, etc.). Then, the changepoint time was defined as the lower bound of the time interval where the fastest slope occurred. Lastly, the early and final slopes are informed by the slope of cognitive decline estimated using a linear mixed model considering the subsets of cognitive measures collected before and after the approximated changepoint, respectively. The linear mixed models used were implemented using the hlme function from the lcmm [23] package (version 1.9.5) to fit mixed effect models on segments of the longitudinal data. Although one of the great advantages of the nlive package is that users do not need to enter initials values, the option *start* allows for manual entry of initials values in a very user-friendly way.

### Overview of the package

The principal function of the package is *nlive*(). This function fits the model requested through the option ”model=1” for SMM, ”model=2” for PMM-abrupt, and ”model=3” for PMM-smooth, and require to take as input a dataset that provides information on the longitudinal outcome of interest, participant ID, time, and predictors (if any). The call to the *nlive* function is:

nlive (model , dataset , ID , outcome , time ,

predictor . all = NULL,

predictor . par1 = NULL,

predictor . par2 = NULL,

predictor . par3 = NULL,

predictor . par4 = NULL,

start = NULL,

traj . marg = FALSE,

traj . marg . group = FALSE)

The first five arguments are mandatory, while all the others have default values. Below is a brief description of the arguments:

- *model:* indicator of the model to fit (1=SMM, 2=abrupt PMM, 3=smooth PMM).
- *dataset:* data frame containing the variables named in *ID, outcome, time, predictor.all*, and *predictor.par*1 to *predictor.par*4.
- *ID*: name of the variable representing the grouping structure specified with ” (e.g., “ID” representing the unique identifier of participants).
- *outcome*: name of the time-varying variable representing the longitudinal outcome specified with ” (e.g., “outcome”)
- *time*: name of the variable representing the timescale specified with ” (e.g., “time”). Can be negative or positive. Note that model 1, SMM, will always report a positive value under the midpoint parameter (e.g. if the time in the data goes from 0 down to −10 and the time of the midpoint is −2, the model will report a midpoint of 2).
- *predictor.all*: optional vector indicating the name of the variable(s) that the four main parameters of the model will be adjusted to (e.g. *predictor.all* = *c*(”*X*1”,”*X*2”)). Default to NULL.
- *predictor.par*1: optional vector indicating the name of the variable(s) that the first main parameter of the model will be adjusted to (e.g. *predictor.all* = *c*(”*X*1”, ”*X*2”)). For model 1, the first parameter = last level. For models 2 and 3, first parameter = intercept. Default to NULL.
- *predictor.par*2: optional vector indicating the name of the variable(s) that the second main parameter of the model will be adjusted to (e.g. *predictor.all* = *c*(”*X*1”, ”*X*2”)). For model 1, the second parameter = initial level. For models 2 and 3, second parameter = slope1 (slope before the change-point). Default to NULL.
- *predictor.par*3: optional vector indicating the name of the variable(s) that the third main parameter of the model will be adjusted to (e.g. *predictor.all* = *c*(”*X*1”,”*X*2”)). For model 1, the third parameter = midpoint. For models 2 and 3, third parameter = slope2 (slope between intercept and changepoint). Default to NULL.
- *predictor.par*4: optional vector indicating the name of the variable(s) that the fourth main parameter of the model will be adjusted to (e.g. *predictor.all* = *c*(”*X*1”, ”*X*2”)). For model 1, the fourth parameter is the Hill slope. For models 2 and 3, the fourth parameter is the changepoint. Default to NULL.
- *start*: optional vector to override the specification of the four initial values for the main parameters. For model 1, the values must be included in the following order: last level, initial level, midpoint, Hill slope. For models 2 and 3, the values must be included in the following order: intercept, slope1 (slope before the changepoint), slope2 (between intercept and the changepoint), and changepoint. Default to NULL.
- *traj.marg*: logical indicating if the marginal estimated trajectories should be plotted. Default to FALSE.
- *traj.marg.group*: name of the grouping variable listed in one of the predictor arguments to plot and contrast the estimated marginal trajectories between two specific groups. If the variable is binary, the trajectories are contrasted between the two groups of interest. If the variable is continuous, the 10^*th*^ and 90^*th*^ percentile values will automatically be considered. Default to NULL.

The list of options that can be set is described in detail in the help files provided with the package (https://CRAN.R-project.org/package=nlive).

## Results

### Performance

We employed a simulation study to evaluate the convergence adequacy of the SMM and PMM models fitted using the SAEM algorithm. For all the models, we examined the convergence rate and the average computation times, after considering an increasing number of individuals (i.e., n=100, 200, and 500) and an increasing number of predictors for each of the 4 parameters (i.e., zero, one, and two covariates). We simulated data close to the longitudinal cognitive trajectories observed before death in ROSMAP [17]. Decedent-specific visit times were generated using a uniform distribution in [−2, 2] months around theoretical annual visits from −24 years to death (year 0) over an average follow-up of 10 years (SD=5); individuals had to have at least 4 cognitive observations.

To evaluate the SMM estimation, the longitudinal cognitive response was generated using a flexible sigmoidal structure. The marginal cognitive trajectory was characterized by an early, progressive decline over time and a moderate acceleration close to time 0, with $\psi_{1}$ (last level) = −1.03, $\psi_{2}$ (initial level) = 0.37, $\psi_{3}$ (midpoint) = −4.0, and $\psi_{4}$ (Hill slope) = 1.69; a variance of 2.13 $\left(\sigma_{\eta_{1}}=1.46\right)$ and 0.26 $\left(\sigma_{\eta_{2}}=0.51\right)$ for the random effects $\eta_{1}$ and $\eta_{2}$, respectively; a correlation of zero between random effects; and an error variance of 0.08 $\left(\sigma_{\epsilon }=0.28\right)$. For the PMM models, the longitudinal cognitive response was generated using a piecewise linear structure with $\psi_{1}$ (last level) = −1.21, $\psi_{2}$ (slope before changepoint) = −0.03, $\psi_{3}$ (slope between intercept and changepoint) = −0.32, and $\psi_{4}$ (changepoint) = −3; a variance of 2.13 $\left(\sigma_{\eta_{1}}=1.70\right)$, 0.26 $\left(\sigma_{\eta_{2}}=0.01\right)$, 2.13 $\left(\sigma_{\eta_{3}}=0.05\right)$, and 0.26 $\left(\sigma_{\eta_{4}}=6.91\right)$ for the random effects *η*_1_, *η*_2_, *η*_3_, and *η*_4_, respectively; a correlation of −0.07 between the random effects *η*_2_ and *η*_3_; and an error variance of 0.08 $\left(\sigma_{\epsilon }=0.28\right)$. For each Scenario, we generated 500 datasets.

**Figure 1:**
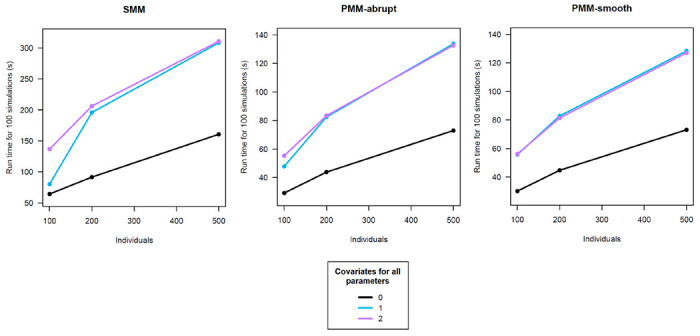
Evolution of computation times with the number of individuals, stratified by the number of covariates considered for all the 4 parameters.

For each model, we found that the convergence rate was excellent (100%). Mean Squared Errors from the average of the estimated marginal cognitive trajectories ranged from 0.07 to 0.02. Figure 1 displays the computation time for a varying sample size stratified by the number of predictors considered for all parameters. We observed that run times generally increased with the sample size and that there is a gap between the models with no covariates and those adjusted. Additionally, we found that compared with PMMs, the SMM model generally required more time to converge, although this time was reasonable on average (see Fig. 1). Of note, the models were fitted on a HP ProBook 400 G6 containing an i7-8565U processor and 16 gigabytes of RAM running R version 4.0.3. Together, these profiling results support that the application of the SAEM algorithm to fit SMM, PMM-abrupt, and PMM-smooth is efficient.

### Example

In this section, we show how the *nlive*() function can be used to fit the SMM, PMM-abrupt, and PMM-smooth models, and we present the main outputs provided by the package. In the context of our motivating application, late-life cognitive decline, each model were fitted using the made-up illustrative sample *dataset* available in the package. Thus, the first step consists in loading *nlive*, which will automatically load *dataset.*

R > library ( nlive )

#### Data

The *dataset* contains 1200 individuals with annual cognitive testing for at least 4 years until death (mean follow-up=7 [SD=5] years); description of the data can be accessed via the command *summary* (*dataset*). On each line, we can read the unique participant identifier (*ID*), the negative retrospective time before death in years (*time*), the repeated values of the composite score of global cognition collected over time (*cognition*), and the age at death of individuals in years; in the natural scale (*ageDeath*) and centered at its mean (*ageDeath* − 90 = *ageDeath*90) for interpretation purposes. The following lines create the continuous *ageDeath*90 variable and display the first lines of *dataset*:

R > dataset $ ageDeath90 <– dataset $ ageDeath – 90

R > head ( dataset )

**Table:** 

	ID	time	cognition	ageDeath	ageDeath90
1	1000	−10.00	0.45	91	1
2	1000	−9.08	0.27	91	1
3	1000	−8.04	0.19	91	1
4	1000	−6.82	0.15	91	1
5	1000	−5.99	0.05	91	1
6	1000	−4.98	0.15	91	1

#### Modeling the SMM

For demonstration purposes, we fit a relatively simple SMM model with all main parameters adjusted for *ageDeath*90. The user only needs to specify the name of the dataframe and the columns containing the participant ID, the response, the timescale, and predictor. We also include arguments to plot the marginal estimated trajectories before death.

R > smm. fit <– nlive (model = 1 , dataset = dataset , ID = ” ID ” ,

+ outcome = ” cognition ” ,

+ time = ” time ” ,

+ predictor . all = c(” ageDeath90 ” ) ,

+ traj . marg = TRUE,

+ traj . marg . group = c(” ageDeath90 ” ))

**Figure 2:**
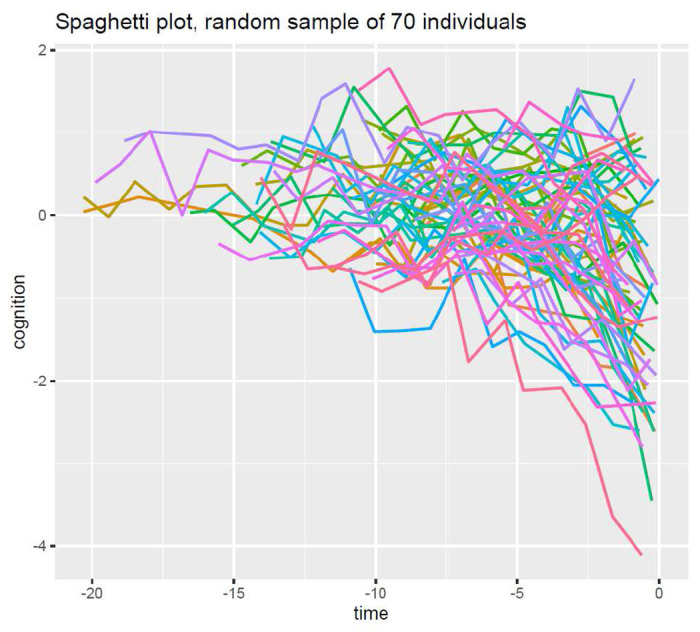
Observed individual trajectories of global cognition in the 20 years before death for 70 individuals randomly selected in the made-up illustrative sample *dataset* available in the *nlive* package.

The first output automatically provided by the package is a spaghetti plot of observed individual cognitive trajectories before death for 70 individuals randomly selected (see Fig. 2). This Figure is produced with the *ggplot2* package [24] and allows to better appreciate the variability of the trajectories within each individual and between individuals over time. Label of axes corresponds to the raw names of covariates. The options *plot.xlabel* and *plot.ylabel* allows the user to specify a character string to define axes x and y, respectively. For example: *plot.xlabel* = *c*(”*Years before death*”). The list of options that can be set is described in detail in the help files provided with the package.

The second output automatically generated corresponds to the general output from *saemix,* which is a summary of the data and the model, followed by the numerical results, including parameter estimates, their standard errors and several statistical criteria [11]. We also added a line that specifies the processing time of the program.

…

**Table:** 

Variance of random effects				

	Parameter	Estimate	SE	CV(%)
last . level	omega2. last . level	1.283	0.0556	4.3
first . level	omega2 . first . level	0.146	0.0071	4.9
covar	cov . last . level . first . level	0.049	0.0143	28.9

Correlation matrix of random effects		

	omega2 . last . level	omega2 . first . level
omega2 . last . level	1.00	0.11
omega2 . first . level	0.11	1.00

Statistical criteria
Likelihood computed by linearisation
−2LL= 9732.152
AIC = 9756.152
BIC = 9817.233
Likelihood computed by importance sampling
−2LL= 9731.84
AIC = 9755.84
BIC = 9816.921

	Parameter	Estimate	SE	p–value
1	last . level	−1.088	0.035	P<.0001
2	beta_ageDeath90 ( last . level )	−0.061	0.004	P<.0001
3	first . level	0.24	0.015	P<.0001
4	beta_ageDeath90 ( first . level )	−0.044	0.002	P<.0001
5	midpoint	2.567	0.034	P<.0001
6	beta_ageDeath90 ( midpoint )	0.031	0.004	P<.0001
7	hill . slope	1.789	0.04	P<.0001
8	beta_ageDeath90 ( hill . slope)	0.007	0.005	0.081
9	error	0.279	0.002	P<.0001

The program took 346.51 seconds

The fitted SMM model indicates that higher age at death was associated with lower cognitive level at baseline (see term beta_ageDeath90(first.level)) and close to death (see term beta_ageDeath90(last.level)). In addition, higher age at death was associated with an earlier half of cognitive decline (see term beta_ageDeath90(midpoint)). However, age at death was not associated with the Hill slope(see term beta_ageDeath90(hill.slope)). It is important to note here that the ”midpoint” and ”hill.slope” parameters have an interpretation in terms of the positive number of years before death as SMM models can only be fitted considering a positive timescale. Negative timescales, like in this example, are automatically converted in the function to positive time for model fitting purposes.

**Figure 3:**
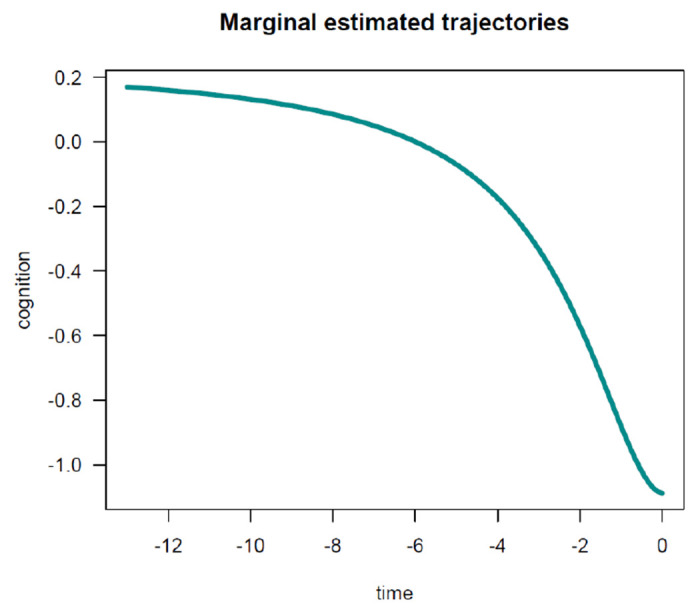
Estimated marginal trajectory of global cognition before death, using the Sigmoidal Mixed Model.

**Figure 4:**
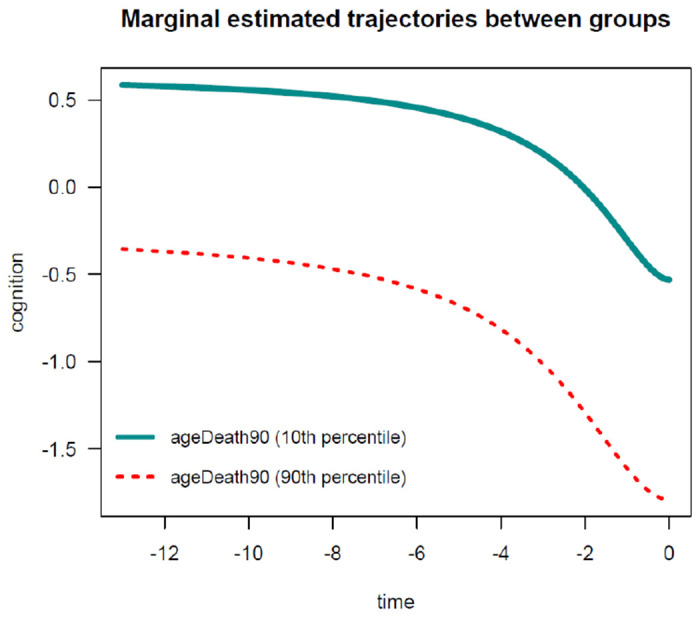
Estimated marginal trajectory of global cognition before death, according to age at death, using the Sigmoidal Mixed Model.

To facilitate the interpretation of the estimated parameters, it is convenient to visualize the estimated average trajectories over time. In *nlive*(), users can easily plot two types of marginal estimated trajectories. First, by setting up the argument *traj.marg* = *T*, the function can provide a graph of the estimated marginal trajectory of global cognition before death in the whole study sample, for the most common profile of covariates (see Fig. 3). In this example, this would represent the most common average age at death (i.e., 90 years). Second, by specifying *traj.marg.group* = *c*(”*ageDeath*90”), the function can provide a plot of estimated marginal trajectories of global cognition contrasted between two groups corresponding to participants in the 10^*th*^ versus 90^*th*^ percentile of the *ageDeath*90 distribution, for the most common profile of covariates (see Fig. 4). Users can manually specify the percentile values using the *traj.marg.group.val* option. For example, *traj.marg.group.val* = *c*(0.25, 0.75) will plot trajectories for the 25^*th*^ and 75^*th*^ percentiles, respectively.

Since *nlive*() fits the models based on the *saemix* package, users can take advantage of many *saemix* functions available to extract specific information from the model object *smm.fit.* Generic functions include, for example, *psi*(*smm.fit*, *type* = ”*mean*”), which extracts the subject-specific predictions; *eta*(*smm.fit*, *type* = ”*mean*”), which extracts the subject-specific random effects; *plot*(*smm.fit*, *plot.type* = ”*convergence*”), which provides the convergence plots; and *plot*(*pmm.abrupt.fit*, *plot.type* = ”*individual.fit*”, *ilist* = *c*(1 : 9), which plots the observed and predicted values of the first nine subject. All the *saemix* functions are available in the online help.

### Modeling the abrupt PMM

For PMM-abrupt, the call of *nlive*() is:

R > pmm. abrupt . fit <– nlive (model = 2, dataset = dataset, ID = ”ID” ,

+ outcome = ” cognition ” ,

+ time = ” time ” ,

+ predictor . all = c(” ageDeath90 ”) ,

+ traj . marg = TRUE,

+ traj . marg .group = c(” ageDeath90 ”))

The general summary output is:

…

**Table:** 

Variance of random effects				

	Parameter	Estimate	SE	CV(%)
last . level	omega2 . last . level	1.07196	4.7 e–02	4.4
slope 1	omega2 . slope 1	0.00062	7.4 e–05	11.9
slope 2	omega2 . slope 2	0.03830	2.0 e–03	5.2
changepoint	omega2 . changepoint	0.58980	7.9 e–02	13.4
covar	cov . slopel . slope 2	0.00378	3.2 e–04	8.4

Correlation matrix of random effects			

	omega2 . last . level	omega2 . slope 1	omega2 . slope 2
omega2 . last . level	1	0.00	0.00
omega2 . slope 1	0	1.00	0.78
omega2 . slope 2	0	0.78	1.00
omega2 . changepoint	0	0.00	0.00
	omega2 . changepoint		
omega2 . last . level	0		
omega2 . slope 1	0		
omega2 . slope 2	0		
omega2 . changepoint	1		

Statistical criteria
Likelihood computed by linearisation
−2LL= 12349.9
AIC = 12377.9
BIC = 12449.16
Likelihood computed by importance sampling
−2LL= 12296.83
AIC = 12324.83
BIC = 12396.1

	Parameter	Estimate	SE	p–value
1	last . level	−1.103	0.031	P<.0001
2	beta_ageDeath90 ( last . level )	−0.062	0.004	P<.0001
3	slope 1	−0.017	0.002	P<.0001
4	beta_ageDeath90 ( slope1 )	−0.0003	0.0004	0.082
5	slope 2	−0.249	0.007	P<.0001
6	beta_ageDeath90 ( slope2 )	−0.001	0.001	0.159
7	changepoint	−4.25	0.048	P<.0001
8	beta_ageDeath90 ( changepoint )	−0.059	0.006	P<.0001
9	error	0.281	0.002	P<.0001

The program took 168.58 seconds

**Figure 5:**
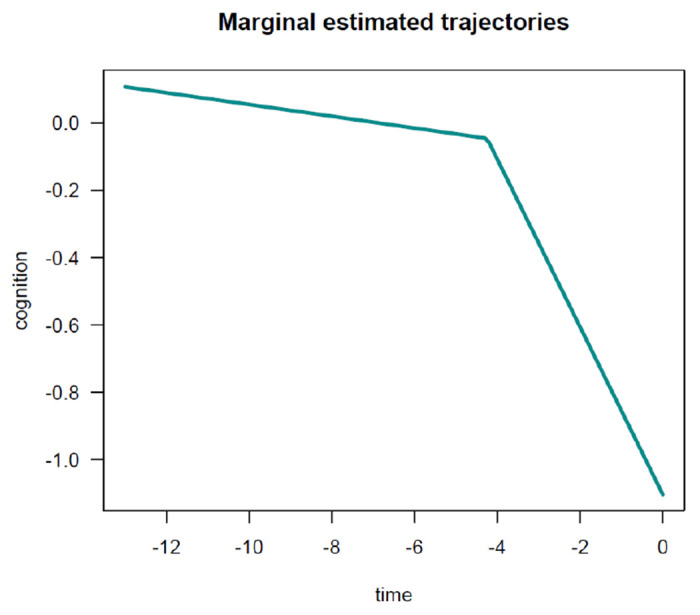
Estimated marginal trajectory of global cognition before death, using the Piecewise Mixed Model with abrupt change.

**Figure 6:**
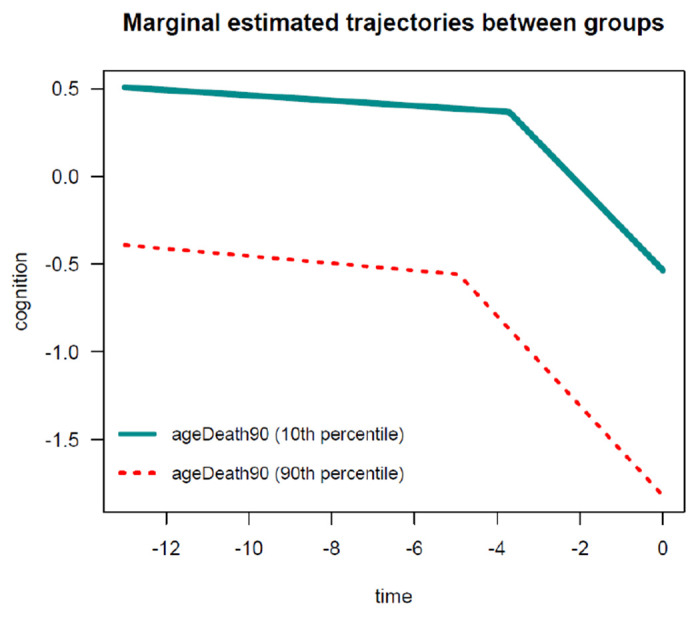
Estimated marginal trajectory of global cognition before death, according to age at death, using the Piecewise Mixed Model with abrupt change.

In this example, for the PMM-abrupt model, we found that each additional year of age at death was associated with worse mean cognitive level close to death (see term beta_ageDeath90(last.level)). In addition, each increment in the age at death was related to an earlier onset of accelerated decline (see term beta_ageDeath90(changepoint)). However, age at death was not related to the preterminal decline (see term beta_ageDeath90(slope1)) or terminal decline (see term beta_ageDeath90(slope2)). The marginal estimated trajectories in the whole study sample and in the 10^*th*^ versus 90^*th*^ percentiles of the age at death distribution are displayed in Figure 5 and Figure 6, respectively.

### Modeling the smooth PMM

For PMM-smooth, the call of *nlive*() is:

R > pmm. smooth .fit <– nlive (model = 3, dataset = dataset, ID = ” ID ” ,

+ outcome = ” cognition ” ,

+ time = ” time ” ,

+ predictor . all = c(” ageDeath90 ”) ,

+ traj .marg = TRUE,

+ traj . marg . group = c(” ageDeath90 ”))

The general summary output is:

…

**Table:** 

Variance of random effects				

	Parameter	Estimate	SE	CV(%)
last . level	omega2 . last . level	1.0699	4 . 7 e–02	4 . 4
slope 1	omega2 . slope 1	0.0006	7 . 3 e–05	12 . 1
slope 2	omega2 . slope 2	0.0377	2 . 0 e–03	5 . 2
changepoint	omega2 . changepoint	0.6037	8 . 1 e–02	13 . 3
covar	cov . slopel . slope2	0.0038	3 . 1 e–04	8 . 3

Correlation matrix of random effects			

	omega2 . last . level	omega2 . slope 1	omega2 . slope2
omega2 . last . level	1	0.00	0.00
omega2 . slope 1	0	1.00	0.79
omega2 . slope 2	0	0.79	1.00
omega2 . changepoint	0	0.00	0.00
	omega2 . changepoint		
omega2 . last . level	0		
omega2 . slope 1	0		
omega2 . slope 2	0		
omega2 . changepoint	1		

Statistical criteria
Likelihood computed by linearisation
−2LL= 12357.39
AIC = 12385.39
BIC = 12456.65
Likelihood computed by importance sampling
−2LL= 12293.15
AIC = 12321.15
BIC = 12392.41

	Parameter	Estimate	SE	p–value
1	last . level	−1.099	0.031	P<.0001
2	beta_ageDeath90 ( last . level )	−0.062	0.004	P<.0001
3	slope 1	−0.017	0.002	P<.0001
4	beta_ageDeath90 (slope 1 )	−0.0003	0.0004	0.082
5	slope 2	−0.246	0.007	P<.0001
6	beta_ageDeath90 ( slope2 )	−0.001	0.001	0.159
7	changepoint	−5.3	0.049	P<.0001
8	beta_ageDeath90 ( changepoint )	−0.058	0.006	P<.0001
9	error	0.281	0.002	P<.0001

The program took 134.25 seconds

**Figure 7:**
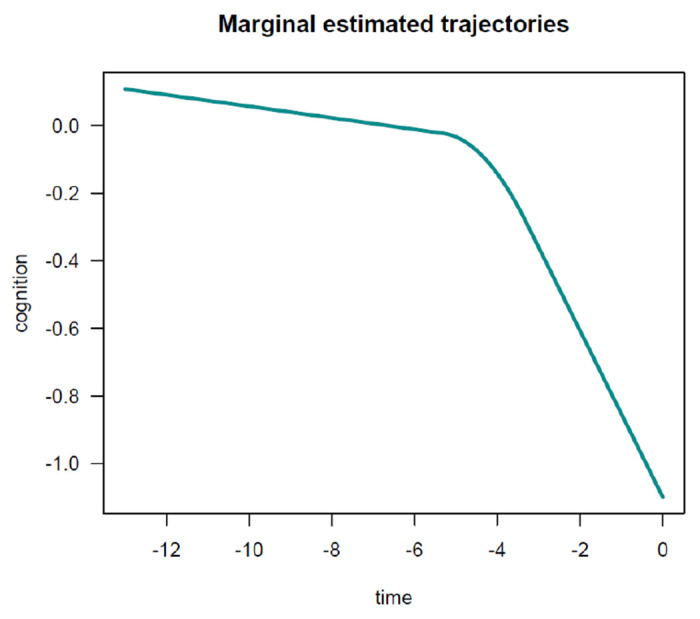
Estimated marginal trajectory of global cognition before death, using the Piecewise Mixed Model with smooth polynomial transition.

**Figure 8:**
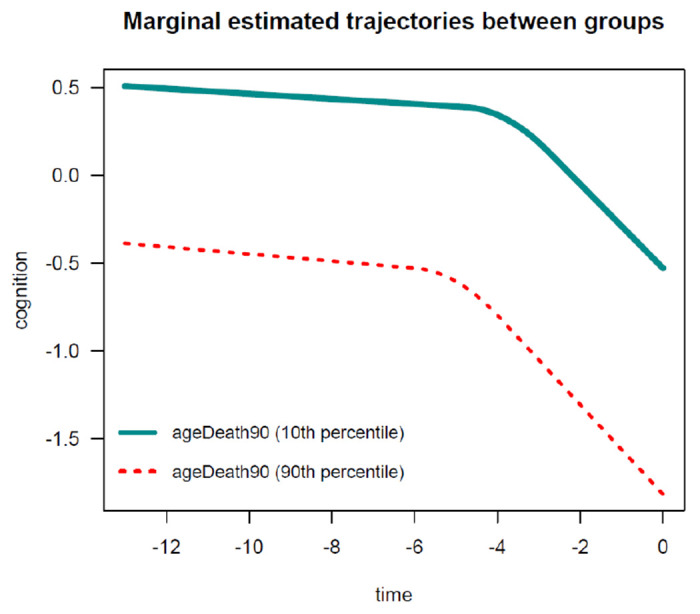
Estimated marginal trajectory of global cognition before death, according to age at death, using the Piecewise Mixed Model with smooth polynomial transition.

In this example, as expected, findings are generally similar to those obtained for the PMM-abrupt model. The main difference is that the estimated changepoint parameter represents here the beginning of the transition period. Marginal estimated trajectories in the whole study sample and according to age at death are displayed in Figure 7 and Figure 8, respectively.

## Concluding remarks

In this work, we introduce a newly developed R package *nlive* to fit three non-linear mixed models for Gaussian longitudinal data: the sigmoidal mixed model (SMM) and two piecewise linear mixed models with a random changepoint (PMM-abrupt and PMM-smooth). The SMM includes 4 parameters, which allow for estimation of early level, half of the decline, Hill slope (the steepness of the curve), and final level of the longitudinal outcome of interest. The two PMM separate the trajectory into two linear phases and allow for estimation of the early slope, changepoint, final slope, and final level. These models were chosen for the implementation as they currently cannot be easily implemented in R and are of importance, especially in aging research. All needed pieces such as functions, covariance matrices, and initials generation were programmed. The *nlive*() function allows fitting these models with one line of code that is intuitive enough to the less sophisticated users. The yielding product has only five mandatory arguments. Options are available to readily accommodate user preferences, including manual specification of starting values or diagnostic plots. It was also designed to help interpretation of the output by providing features such as annotated output, warnings (e.g. small sample, number of covariates), and graphs.

This package is the first to provide a seamless user interface to fit the Sigmoidal Mixed Effect Model. Some packages in R can fit the Sigmoid curve but not the mixed effect model. Most of these packages focus on dose-response optimization and curve-fitting [25] such as *qpcR* [26], *grofit* [27], *FlexParamCurve* [28], *drfit* [29], and *MCPMod* [30] or aim to automated fitting and classify multiple curves [25]. Although this is not the first package to provide an interface to fit the PMM, the *nlive* includes the more recent developments in the model structure and the likelihood maximization algorithm. The smooth PMM implemented is based on the polynomial transition that was demonstrated to have improved properties over to the Bacon-Wats. PMM models were also reparameterized which allows the interpretation based on the estimated value at time zero and not a projection to zero from the first and more distant slope. Here we build upon recently developed tools in R such as the *saemix* package that utilizes the Stochastic Approximation EM-based algorithm, shown in several tests to have a better convergence rate than the Maximum Likelihood. All models fit with the same algorithm. Extensive testing of functionality was already performed for *saemix* development. In this interface, however, convergence adequacy was tested given the particular complexity of these models. Overall the convergence rate was high, the time was reasonable, and the bias was low.

The motivation of this package was aging research including biomarkers of the Alzheimer’s pathological cascade (a.k.a. Jack curves) [31], natural history of cognition [4, 32, 33], retesting effect [34, 35], and terminal decline [36, 37]. These models, however, are non-specific and the nlive can be used in a wide variety of fields. Many processes were demonstrated to follow a sigmoid trajectory over time (a.k.a. 3 to 5 parameters logistic, Hill, Langmuir, Langmuir–Hill, and Hill–Langmuir equation). Such processes are found in agriculture [1], pharmacology [2] and marketing [3], to cite a few. Similarly, many processes that are initially linear may have an unknown change that may modify the trajectory. Such processes are found in a wide variety of fields from environmental sciences [38] to engineering [39].

In conclusion, we hope that this very user-friendly package will encourage the adoption of more sophisticated models for longitudinal data by the R community, with varying degrees of experience. Although illustrated in the context of cognitive aging, the package can be used in a wide variety of applications.

## Data Availability

The R package nlive can be installed directly using install.packages(”nlive”) in an R console. Archived versions are available from the Comprehensive R Archive Network (CRAN) at https://CRAN.R-project.org/package=nlive. The made-up illustrative dataset analysed during the current study is bundled with the R package nlive, and can be accessed by running the command data(dataset, package = ”nlive”).

## Abbreviations

CRAN: the comprehensive R archive newtwork
PMM: piecewise mixed model
SAEM: stochastic approximation expectation maximization
SMM: sigmoidal mixed model
